# Store depletion induces Gαq-mediated PLCβ1 activity to stimulate TRPC1 channels in vascular smooth muscle cells

**DOI:** 10.1096/fj.15-280271

**Published:** 2015-10-14

**Authors:** Jian Shi, Francesc Miralles, Lutz Birnbaumer, William A. Large, Anthony P. Albert

**Affiliations:** *Vascular Biology Research Centre, Institute of Cardiovascular and Cell Sciences, and ^†^Institute of Medical and Biomedical Education, St. George’s, University of London, London, United Kingdom; and ^‡^Laboratory of Neurobiology, National Institute of Environmental Health Sciences, Research Triangle Park, North Carolina, USA

**Keywords:** electrophysiology, PLC activity, Ca^2+^ signaling, phosphoinositol signaling

## Abstract

Depletion of sarcoplasmic reticulum (SR) Ca^2+^ stores activates store-operated channels (SOCs) composed of canonical transient receptor potential (TRPC) 1 proteins in vascular smooth muscle cells (VSMCs), which contribute to important cellular functions. We have previously shown that PKC is obligatory for activation of TRPC1 SOCs in VSMCs, and the present study investigates if the classic phosphoinositol signaling pathway involving Gαq-mediated PLC activity is responsible for driving PKC-dependent channel gating. The G-protein inhibitor GDP-β-S, anti-Gαq antibodies, the PLC inhibitor U73122, and the PKC inhibitor GF109203X all inhibited activation of TRPC1 SOCs, and U73122 and GF109203X also reduced store-operated PKC-dependent phosphorylation of TRPC1 proteins. Three distinct SR Ca^2+^ store-depleting agents, 1,2-bis(2-aminophenoxy)ethane-*N,N,N′,N*′-tetraacetic acid acetoxymethyl ester, cyclopiazonic acid, and *N*,*N*,*N′*,*N′*-tetrakis(2-pyridylmethyl)ethane-1,2-diamineed, induced translocations of the fluorescent biosensor GFP-PLCδ1-PH from the cell membrane to the cytosol, which were inhibited by U73122. Knockdown of PLCβ1 with small hairpin RNA reduced both store-operated PLC activity and stimulation of TRPC1 SOCs. Immunoprecipitation studies and proximity ligation assays revealed that store depletion induced interactions between TRPC1 and Gαq, and TRPC1 and PLCβ1. We propose a novel activation mechanism for TRPC1 SOCs in VSMCs, in which store depletion induces formation of TRPC1-Gαq-PLCβ1 complexes that lead to PKC stimulation and channel gating.—Shi, J., Miralles, F., Birnbaumer, L., Large, W. A., Albert, A. P. Store depletion induces Gαq-mediated PLCβ1 activity to stimulate TRPC1 channels in vascular smooth muscle cells.

Plasma membrane store-operated channels (SOCs) are physiologically induced by extracellular agents, which stimulate the classic phosphoinositol signaling pathway composed of Gαq-coupled receptors, PLC activation, phosphatidyinositol 4,5-bisphosphate (PIP_2_) hydrolysis, and generation of inositol 1,4,5-trisphosphate (IP_3_) and diacylglyercol (DAG) that leads to IP_3_-mediated depletion of endoplasmic/sarcoplasmic reticulum (SR) Ca^2+^ stores. In vascular smooth muscle cells (VSMCs), SOCs have been proposed to mediate Ca^2+^ entry pathways, which regulate cellular functions such as contraction, proliferation, and migration that are linked to regulation of vascular tone, and the development of hypertension and atherosclerosis (1–3). Consequently, understanding molecular mechanisms involved in gating SOCs is an important objective in vascular physiology.

It is now firmly established that the archetypal store-operated current *I*_crac_, which is characterized by high Ca^2+^ permeability, pronounced inward rectification, and a unitary conductance in order of fS, is formed by Orai1 channel proteins (4–7). Moreover, it is recognized that Ca^2+^ store depletion induces oligomerization and translocation of the endoplasmic/SR Ca^2+^ sensor STIM1 to the plasma membrane where it induces Orai1 channel opening (4–7). It is also apparent that many cell types express SOCs, which have much lower Ca^2+^ permeabilities, relatively linear current-voltage (*I/V*) relationships, and larger unitary conductances compared to Orai1-mediated *I*_crac_. These SOCs are proposed to be mediated by the canonical transient receptor potential (TRPC) family of Ca^2+^-permeable nonselective cation channel proteins (TRPC1–C7) (8, 9), with TRPC1, TRPC3, and TRPC4 subtypes particularly implicated in composing SOCs. Because TRPC subunits form heteromeric channel structures, it is likely that there are many distinct TRPC-mediated SOCs, which are formed of diverse TRPC subunit arrangements (9).

The present study investigates the role of TRPC1 in regulating SOCs in VSMCs. A significant problem with defining TRPC1 channels as SOCs, as with all TRPC channels, has been determining how depletion of Ca^2+^ stores is coupled to channel gating. Several ideas have outlined possible activation mechanisms of TRPC1 SOCs, including direct gating by STIM1 through electrostatic and protein-protein interactions and store-operated STIM1/Orai1-mediated Ca^2+^ entry increasing trafficking of TRPC1 proteins to the plasma membrane (8–13). The present study proposes the idea that store-operated G-protein-PLC-PKC activities drive activation of TRPC1 channels in VSMCs.

Several studies have described SOCs in VSMCs from several different vascular preparations, which have relatively linear *I/V* relationships and unitary conductances of ∼2 pS, and are proposed to be mediated by a heteromeric TRPC1/C5 molecular template (2, 14–22). Importantly, transgenic mouse studies have indicated that TRPC1 proteins are the essential subunits that confer channel gating by store depletion, and therefore, these heteromeric TRPC1/C5 structures in VSMCs are often termed TRPC1 SOCs (22). We have shown that PKC-dependent phosphorylation of TRPC1 proteins is obligatory for activation of TRPC1 SOCs because this event is critical for channel opening by PIP_2_ (15, 17, 20, 22–25). It is thought that in unstimulated VSMCs, TRPC1 SOCs remain closed due to interactions between TRPC1 and the PIP_2_-binding protein myristoylated alanine-rich C-kinase substrate (MARCKS), with MARCKS acting as a localized PIP_2_ buffer to prevent channel activation (25). PKC-dependent phosphorylation of TRPC1 by store depletion causes dissociation of MARCKS from TRPC1 and also MARCKS to release PIP_2_, which enables this phospholipid to act as the gating ligand (25). It is currently not understood how store depletion couples to PKC activity, and this question forms the focus of the current work.

The present study reveals for the first time that Gαq-mediated PLCβ1 activity is activated by Ca^2+^ depletion within SR Ca^2+^ stores in VSMCs. This activation mechanism is associated with formation and stimulation of store-operated Gαq-PLCβ1-TRPC1 complexes, which induce PKC-dependent phosphorylation of TRPC1 subunits and channel opening. These results are likely to be important in functioning of VSMCs and also may have more widespread importance because phosphoinositol signaling and TRPC1 channels are ubiquitously expressed among cell types.

## MATERIALS AND METHODS

### Cell isolation

New Zealand white rabbits (2–3 kg; Highgate Farm, Louth, United Kingdom) were killed using intravenous sodium pentobarbitone (120 mg/kg), and mice were killed using cervical dislocation according to the UK Animals Scientific Procedures Act of 1986. Portal veins or second-order mesenteric arteries were dissected free and cleaned of fat, connective tissue, and endothelium in physiologic salt solution containing 126 mM NaCl, 6 mM KCl, 10 mM glucose, 11 mM 4-(2-hydroxyethyl)-1-piperazineethanesulfonic acid (HEPES), 1.2 mM MgCl_2_, and 1.5 mM CaCl_2_ (pH adjusted to 7.2 using 10 M NaOH). Vessels were enzymatically dispersed into single VSMCs as previously described (19, 21).

### Electrophysiology

Whole-cell and single-channel cation currents were made with an AXOpatch 200B amplifier (Axon Instruments, Union City, CA, USA) at room temperature (20–23°C) as described previously (21). Whole-cell currents were filtered at 1 kHz (−3 dB, low-pass 8-pole Bessel filter, Frequency Devices model LP02; Scensys, Aylesbury, United Kingdom) and sampled at 5 kHz (Digidata 1322A and pCLAMP 9.0 software; Molecular Devices, Sunnyvale, CA, USA). Whole-cell *I/V* relationships were obtained by applying 750 ms duration voltage ramps from +100 to −150 mV every 30 s from a holding potential of 0 mV. Single-channel currents were filtered between 0.1 and 0.5 kHz and acquired at 1–5 kHz. Single-channel *I/V* relationships were evaluated by manually altering the holding potential of −80 mV between −120 and +120 mV. For single-channel analysis, single-channel current amplitudes were calculated from idealized traces of ≥60 s in duration using the 50% threshold method and analyzed using pCLAMP 9.0 software. Events lasting for <6.664 ms [2× rise time for a 100 Hz (−3 dB) low-pass filter] were excluded from analysis to maximize the number of channel openings reaching their full current amplitude. Open probability was used as a measure of channel activity and was calculated automatically by pCLAMP 9. Single-channel current amplitude histograms were plotted from the event data of the idealized traces with a 0.01 pA bin width. Amplitude histograms were fitted using gaussian curves with peak values corresponding to channel open levels. Mean channel amplitudes at different membrane potentials were plotted, and *I/V* relationships were fitted by linear regression with the gradient determining conductance values. Figures were prepared using MicroCal Origin 6.0 software (MicroCal Software, Northampton, MA, USA), in which inward single-channel openings are shown as downward deflections.

### Primary cell culture

VSMCs were seeded into culture plates, maintained using DMEM/F-12 medium containing 1% serum, and incubated at 37°C in 95% O_2_: 5% CO_2_ at 100% humidity for up to 7 d. In 1% serum, VSMCs maintained their contractile phenotype (see Supplemental Fig. S1*C*). Single TRPC1 channel currents evoked by store depletion and other previously described stimulators (10–14) were similar in freshly dispersed and primary cultured cells (Supplemental Fig. S1*B*), which suggests that compensatory changes to channel properties were unlikely in these cell culture conditions.

### Imaging of GFP-PLCδ-PH-mediated signals

VSMCs were transfected with GFP-PLCδ-PH (plasmid identification, 21179; Addgene, Cambridge, MA, USA) using Nucleofector according to the manufacturer’s instructions (Amaxa Biosystems, Gaithersburg, MD, USA). A total of 0.2–0.4 μg plasmid DNA was added to 1 × 10^5^ cells resuspended in 20 μl Nucleofector solution, and cells were kept in primary cell culture conditions for up to 3 d. Transfected cells were imaged using a Zeiss LSM 510 laser-scanning confocal microscope and associated software (Carl, Jena, Germany). Excitation was produced by 488/405 nm lasers and delivered *via* a Zeiss Apochromat 63 oil-immersion objective (numerical aperture, 1.4). Two-dimensional images cut horizontally through approximately the middle of the cells were captured (1024 × 1024 pixels). Final images were produced using PowerPoint (Microsoft XP; Microsoft, Redmond, WA, USA). To prevent contraction of VSMCs following pretreatment with noradrenaline, which precludes accurate imaging of GFP-PLCδ-PH signals (see Supplemental Fig. S1*C*), we bathed cells in 1 μM wortmannin to inhibit myosin light-chain kinase.

### Knockdown of PLCβ1

We used a lentiviral-mediated delivery of pLKO.1-puro–based small hairpin RNA (shRNA) expression plasmids purchased from Sigma-Aldrich (Gillingham, United Kingdom) to knock down PLCβ1. Transduced VSMCs were selected with 2.5 μg/ml puromycin (Invitrogen–Life Technologies, Carlsbad, CA, USA) for 2 d prior to performing immunoblots. PLCβ1 shRNA1 and shRNA2 target PLCβ1 RNA at 5′-GCAGATAAACATGGGCATGTA-3′ and 5′-GCTGTCTTTGTCTACATAGAA-3′, respectively. Scrambled shRNA sequences were used as controls.

### Proximity ligation assay

Freshly isolated VSMCs were studied using the Duolink *in situ* PLA detection kit 563 (Olink, Uppsala, Sweden). Cells were adhered to coverslips, fixed in PBS containing 4% paraformaldehyde for 15 min, and permeabilized in PBS containing 0.1% Triton X-100 for 15 min. Cells were blocked for 1 h at 37°C in blocking solution and incubated overnight at 4°C with anti-TRPC1, anti-Gαq, and anti-PLCβ1 antibodies (all at 1:200) in antibody diluent solution. Cells were labeled with combinations of either anti-goat Plus/anti-rabbit Minus or anti-goat PLUS/anti-mouse Minus depending on animal species used for 1 h at 37°C. Hybridized oligonucleotides were ligated for 30 min at 37°C prior to amplification for 100 min at 37°C. Red fluorescently labeled oligonucleotides were then hybridized to rolling circle amplification products and visualized using a confocal LSM 510.

### IP_3_ ELISA

Cells or tissues were quickly lysed or homogenized on ice. IP_3_ production determinations were performed with a rabbit IP_3_ ELISA kit (BlueGene Biotech, Shanghai, China) following the manufacturer’s instructions. The data were reported as picograms of IP_3_ per milligrams of total cell lysate protein.

### Immunoprecipitation and Western blot

Freshly isolated vessel segments or primary cultured cells were lysed by RIPA buffer and then transferred to a microcentrifuge tube (VWR, Lutterworth, United Kingdom). Total cell lysate protein was extracted and immunoprecipitated using antibodies raised against targeted proteins with an EMD Millipore Catch and Release Kit (EMD Millipore, Billerica, MA, USA) followed by 1-dimensional protein gel electrophoresis (15–20 μg total protein per lane). Separated proteins were transferred onto PVDF membranes and then membranes were incubated with the primary antibodies overnight at 4°C. Visualization was performed with a horseradish peroxidase-conjugated secondary antibody (80 ng/ml) and ECL reagents (Pierce Biotechnology, Inc., Rockford, IL, USA) for 1 min and exposure to photographic films. Band intensities were calculated using Image Studio software (Li-Cor Biosciences, Cambridge, United Kingdom) and then were normalized to control bands. Data shown represent findings from ≥3 different animals.

### Immunocytochemistry

Freshly isolated VSMCs were fixed with 4% paraformaldehyde (Sigma-Aldrich) for 10 min, washed with PBS, and permeabilized with PBS containing 0.1% Triton X-100 for 20 min at room temperature. Cells were incubated with PBS containing 1% bovine serum albumin for 1 h at room temperature and then were incubated with primary antibodies in PBS containing 1% bovine serum albumin overnight at 4°C. In control experiments, cells were incubated without the primary antibody. The cells were washed and incubated with secondary antibodies conjugated to a fluorescent probe. Unbound secondary antibodies were removed by washing with PBS, and nuclei were labeled with DAPI mounting medium (Sigma-Aldrich). Cells were imaged using a Zeiss LSM 510 laser-scanning confocal microscope. The excitation beam was produced by an argon (488 nm) or helium/neon laser (543 and 633 nm) and delivered to the specimen *via* a Zeiss Apochromat ×63 oil-immersion objective (numerical aperture, 1.4). Emitted fluorescence was captured using LSM 510 software (release 3.2; Carl Zeiss). Two-dimensional images cut horizontally through approximately the middle of the cells were captured (1024 × 1024 pixels). Raw confocal imaging data were processed and analyzed using Zeiss LSM 510 software. Final images were produced using PowerPoint (Microsoft XP).

### Bathing and patch pipette solutions

In whole-cell recording experiments, the external solution was composed of 135 mM Na-methanesulfonate, 10 mM CsCl, 1.2 mM MgSO_4_, 10 mM HEPES, 20 mM CaCl_2_, 10 mM glucose, 0.005 mM nicardipine, 0.1 mM 4,4-diisothiocyanostilbene-2,2-disulfonic acid, and 0.1 mM niflumic acid, adjusted to pH 7.4 with NaOH. The patch pipette solution contained 145 mM Cs-methanesulfonate, 20 mM 1,2-bis(2-aminophenoxy)ethane-*N,N,N′,N′*-tetraacetic acid (BAPTA), 8 mM MgCl_2_, and 10 mM HEPES, adjusted to pH 7.2 with CsOH. Under these conditions, voltage-dependent Ca^2+^ channels and Ca^2+^-activated and swell-activated Cl^−^ conductances are blocked allowing cation conductances to be recorded in isolation.

In cell-attached patch experiments, the membrane potential was set to 0 mV by perfusing cells in a KCl external solution containing 126 mM KCl, 1.5 mM CaCl_2_, 10 mM HEPES, and 11 mM glucose (pH adjusted to 7.2 with 10 M KOH). A total of 5 μm nicardipine was included to prevent smooth muscle cell contraction by blocking Ca^2+^ entry through voltage-dependent Ca^2+^ channels.

The patch pipette solution used for both cell-attached and inside-out patch recording (extracellular solution) was K^+^-free and contained 126 mM NaCl, 1.5 mM CaCl_2_, 10 mM HEPES, 11 mM glucose, 10 mM TEA, 5 mM 4-AP, 0.0002 mM iberiotoxin, 0.1 mM 4,4-diisothiocyanostilbene-2,2-disulfonic acid, 0.1 mM niflumic acid, and 0.005 mM nicardipine (pH adjusted to 7.2 with NaOH). The bath solution used for inside-out patch recording (intracellular solution) contained 18 mM CsCl, 108 mM Cs-aspartate, 1.2 mM MgCl_2_, 10 mM HEPES, 11 mM glucose, 1 mM Na_2_ATP, and 0.2 mM NaGTP (pH adjusted to 7.2 with Tris). Free [Ca^2+^]_i_ was set at 100 nM by adding 0.48 mM CaCl_2_ plus 1 mM 1,2-bis(2-aminophenoxy)ethane-*N,N,N′,N′*-tetraacetic acid acetoxymethyl ester (BAPTA-AM) using EqCal software (Biosoft, Cambridge, United Kingdom).

### Reagents

Drugs were from Sigma-Aldrich unless otherwise stated. Rabbit anti-TRPC1 antibody was generated by GenScript (Piscataway, NJ, USA) using peptide sequences from a previously characterized putative extracellular region (14, 26). Goat anti-TRPC1 (sc-15055), mouse anti-Gαq (sc-136181), mouse anti-P-Thr (sc-5267), mouse anti-P-Ser (sc-81514), goat anti-PLCβ1 (sc-31755), mouse anti-PLCβ1 (sc-5291), and mouse anti-PLCγ1 (sc-7290) antibodies were obtained from Santa Cruz Biotechnology (Dallas, TX, USA). Anti-Gαq/11 (06-709), anti-Gαi1-2 (06-236), and anti-Gαi3 (06-270) antibodies were from EMD Millipore. All secondary antibodies were obtained from Santa Cruz Biotechnology. Alexa Fluor 488-conjugated donkey anti-rabbit antibodies and Alexa Fluor 546-conjugated donkey anti-mouse antibodies were from Thermo Fisher Scientific (Waltham, MA, USA). Mouse anti-β-actin antibody (A1978) was obtained from Sigma-Aldrich. All other drugs were purchased from Sigma-Aldrich or Tocris Bioscience (Abingdon, United Kingdom). Agents were dissolved in distilled H_2_O or 0.1% DMSO. DMSO alone had no effect on whole-cell currents or single-channel activity.

### Statistical analysis

This was performed using paired (comparing the effects of agents on the same cell) or unpaired (comparing the effects of agents between cells) Student’s *t* tests with the level of significance set at a value of *P* < 0.05.

## RESULTS

### Activation of TRPC1 channels involves Gαq, PLC, and PKC activities

**Figure 1.**
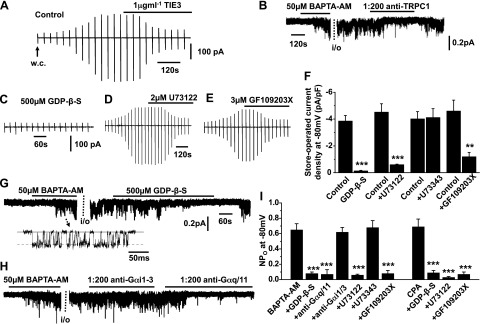
G-protein, PLC, and PKC activities mediate TRPC1 SOCs. *A*) Representative recording shows development of a store-operated whole-cell cation current following break-in into the whole-cell configuration (w.c.), which was inhibited by bath application of the external-acting TRPC1 antibody TIE3. Vertical deflections represent currents evoked by voltage ramps from +100 to −150 mV (750 ms duration) every 30 s from a holding potential of 0 mV. *B*) Representative trace shows that BAPTA-AM-evoked single cation channel activity in cell-attached patches held at −80 mV was maintained following patch excision into the inside-out configuration (i/o) and inhibited by bath application of an internal-acting TRPC1. *C*) Trace shows that development of a store-operated whole-cell TRPC1 current was prevented by inclusion of GDP-β-S in the patch pipette solution. *D*, *E*) Store-operated whole-cell TRPC1 currents were inhibited by bath applications of U731222 (*D*) or GF109203X (*E*). *F*) Mean data show the inhibitory effects of GDP-β-S, U73122, and GF109203X on store-operated whole-cell TRPC1 current densities at −80 mV (each data set is *n* = 6). ***P < 0.001. *G*, *H*) Original recording traces show that BAPTA-AM-evoked single cation channel activity in cell-attached patches held at −80 mV was inhibited by GDP-β-S or a mixture of anti-Gαq and anti-Gα11 antibodies to the cytosolic surface of inside-out patches (*G*), whereas a mixture of anti-Gαi1/2 and anti-Gαi3 antibodies had no effect (*H*). *I*) Mean data show inhibitory actions of GDP-β-S, anti-Gαq/11 antibodies, U73122, and GF109203X on BAPTA-AM-evoked TRPC1 channel activity (each data set is *n* = 6). **P < 0.01; *** *P* < 0.001.

In our initial experiments, we confirmed that SOCs recorded in the present study are mediated by TRPC1 subunits using anti-TRPC1 antibodies as blocking agents. **Figure 1*A*** shows that passive depletion of internal Ca^2+^ stores following cell dialysis with a patch pipette solution containing 20 mM BAPTA and no added Ca^2+^ evoked whole-cell cation currents with relative linear *I/V* relationships and *E*_rev_ of ∼+20 mV, which are similar properties to store-operated TRPC1 currents previously described in VSMCs (22). In addition, Fig. 1*B* and Supplemental Fig. S1*B* show that bath application of 50 µM BAPTA-AM, a cell-permeable Ca^2+^ chelator, activated single-channel activity in cell-attached patches with a unitary conductance of ∼2 pS; again, these properties are similar to those previously shown for single TRPC1 SOCs in VSMCs (15, 17, 18, 20, 22). Complementary to these findings, Fig. 1*A* shows that bath application of 1 μg/ml^−1^ of TIE3, an extracellular-acting anti-TRPC1 antibody (14, 26), inhibited mean peak whole-cell current densities from −4.21 ± 0.63 pA/pF to 1.34 ± 0.22 pA/pF (*n* = 6) at −80 mV. Figure 1*B* also shows that BAPTA-AM-evoked channel activity, maintained following excision of cell-attached patches into the inside-out configuration, was inhibited by bath application of 1:200 dilution of an intracellular-acting anti-TRPC1 antibody to the cytosolic surface of patches, with mean open probability values reduced from 0.64 ± 0.06 to 0.16 ± 0.03 (*n* = 7) at −80 mV.

It is well known that Gαq-mediated PLC activity and production of DAG lead to PKC stimulation, and we have shown that Gαq-coupled receptor agonists and DAG analogs evoke PKC-dependent activation of TRPC1 channels in VSMCs (15, 21, 25). We therefore examined if Gαq and PLC activities are also required for activation of TRPC1 channels by store depletion in freshly isolated rabbit portal vein VSMCs using well-characterized pharmacologic inhibitors of G proteins, PLC, and PKC on store-operated whole-cell and single-channel TRPC1 currents.

Inclusion of 500 µM GDP-β-S, a cell-impermeable G-protein inhibitor, in the patch pipette solution prevented development of store-operated whole-cell TRPC1 currents (Fig. 1*C*, *F* and Supplemental Fig. S1*A*). In addition, bath applications of 2 µM U73122, a PLC inhibitor, and 3 µM GF109203X, a PKC inhibitor, greatly inhibited store-operated whole-cell TRPC1 currents by >75% at all membrane potentials tested (Fig. 1*D*–*F* and Supplemental Fig. S1*A*). The inactive analog of U73122, U73343 at 2 μM concentration, had no effect on store-operated whole-cell TRPC1 currents (Fig. 1*F*).

Figure 1*G*, *I* shows that bath applications of 500 µM GDP-β-S, 2 μM U73122, and 3 μM GF109203X to the cytosolic surface of inside-out patches suppressed BAPTA-AM-evoked TRPC1 channel activity by >85% at −80 mV. Moreover, a mixture of anti-Gαq and anti-Gα11 antibodies at 1:200 dilutions inhibited BAPTA-AM-evoked TRPC1 channel activity in inside-out patches by >85% at −80 mV (Fig. 1*G*–*I*). In contrast, a mixture of anti-Gαi1/2 and anti-Gαi3 antibodies at 1:200 dilutions had no effect on TRPC1 channel activity (Fig. 1*H*, *I*). Stimulation of single TRPC1 channel activities by 10 µM cyclopiazonic acid (CPA), an SR Ca^2+^-ATPase inhibitor, was also suppressed by 500 µM GDP-β-S, 2 µM U73122, and 3 µM GF109203X by >85% at −80 mV (Fig. 1*I*).

**Figure 2.**
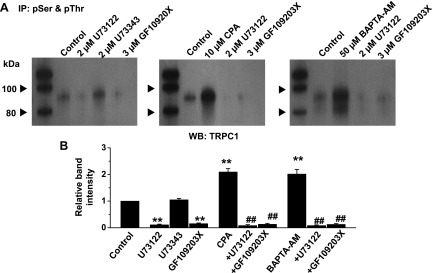
PLC and PKC mediate store-operated phosphorylation of TRPC1 proteins. *A*) Coimmunoprecipitation (IP) of freshly isolated rabbit portal vein tissue lysates with anti-phosphorylated serine (pSer) and threonine (pThr) antibodies followed by Western blotting (WB) with an anti-TRPC1 antibody shows that constitutive TRPC1 phosphorylation was reduced by pretreatment with U73122 or GF109203X, but not by U73343 (left panel). Pretreatment with CPA (middle panel) or BAPTA-AM (right panel) increased phosphorylation of TRPC1, which were inhibited by coapplication of U73122 or GF109203X. *B*) Mean relative band intensities normalized to control bands of data (*n* = 3 different tissue lysate preparations). **P < 0.01 *vs*. control; ^##^*P* < 0.01 *vs*. CPA or BAPTA-AM.

Our previous data indicate that PKC-dependent phosphorylation of TRPC1 proteins is pivotal for activation of TRPC1 SOCs (20, 25), and therefore, we studied if PLC activity is involved in this pathway. Immunoprecipitation of freshly isolated rabbit portal vein vessel lysates with a mixture of anti-phosphorylated serine and anti-phosphorylated threonine antibodies followed by Western blotting with an anti-TRPC1 antibody revealed that TRPC1 proteins displayed a low level of constitutive phosphorylation, which was inhibited by pretreatment with 2 µM U73122 or 3 µM GF109203X (**Fig. 2*A***, ***B***, left panel). Moreover, pretreatment of vessels with 10 µM CPA (Fig. 2*A*, middle panel) or 50 µM BAPTA-AM (Fig. 2*A*, right panel) for 10 min increased phosphorylation of TRPC1 proteins by ∼2-fold, which were reduced by coapplication of 2 µM U73122 or 3 µM GF209203X (Fig. 2*B*). In control experiments, pretreatment of vessels with BAPTA-AM, CPA, U73122, or GF109203X did not alter TRPC1 expression levels (Supplemental Fig. S2*A*).

These findings provide pharmacologic evidence that store depletion is coupled to Gαq-mediated PLC activity and that this pathway induces PKC-dependent phosphorylation of TRPC1 proteins, which is important for stimulation of TRPC1 channels.

### PLCβ1 mediates TRPC1 SOCs in VSMCs

**Figure 3.**
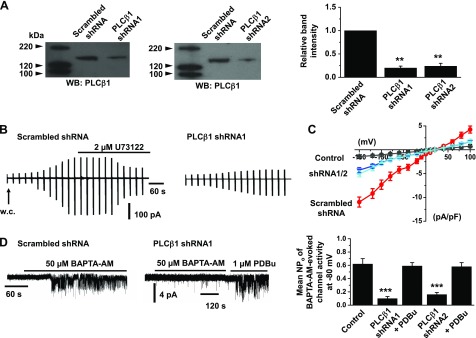
Activation of TRPC1 SOCs is mediated by PLCβ1. *A*) Western blots and mean data confirm that 2 different PLCβ1 shRNA sequences (shRNA1 and shRNA2) reduced PLCβ1 expression (*n* = 3 primary cell culture preparations). **P < 0.01. *B*) Representative traces show that peak amplitude of store-operated whole-cell TRPC1 currents was greatly reduced following transduction of cells with PLCβ1 shRNA1 compared to scrambled shRNA sequences. In the presence of scrambled shRNA, store-operated whole-cell currents were inhibited by U73122. *C*) Mean *I/V* relationships show that PLCβ1 knockdown with shRNA1 and shRNA2 reduced store-operated TRPC1 currents (*n* = 6). *D*) Representative recordings and mean data show that BAPTA-AM-evoked TRPC1 SOC activities were reduced by both PLCβ1 shRNA1 and shRNA2 sequences compared to scrambled shRNA, but this did not affect channel activation by phorbol 12,13-dibutyrate (PDBu) (*n* = 7). ***P < 0.001.

Previous studies have stated that PLCβ1, a PLC isoform, is involved in activation of TRPC channels (27–29), and therefore, we investigated if PLCβ1 contributes to PLC-mediated stimulation of TRPC1 SOCs in VSMCs. Western blot studies showed that PLCβ1 protein is expressed in primary cultured rabbit portal vein VSMCs and that PLCβ1 shRNAs reduced PLCβ1 expression by ∼75% compared to scrambled shRNA sequences (**Fig. 3*A***). In control experiments, PLCβ1 knockdown did not alter TRPC1, Gαq, and β-actin expression levels (Supplemental Fig. S2*B*). It should also be noted that primary cultured VSMCs expressed SOCs with similar single-channel properties and activation mechanisms as TRPC1 SOCs present in freshly dispersed VSMCs (Supplemental Fig. S1*B*). Moreover, primary cultured VSMCs maintained in 1% fetal calf serum for 3–7 d displayed a contractile phenotype (Supplemental Fig. S1*C*).

In VSMCs expressing scrambled shRNA, passive store depletion activated whole-cell TRPC1 currents, which were inhibited by bath application of 2 µM U73122 (Fig. 3*B*). In contrast, treatment of VSMCs with PLCβ1 shRNAs greatly reduced the development of store-operated whole-cell TRPC1 currents at all membrane potentials tested (Fig. 3*B*, *C*). Furthermore, PLCβ1 knockdown reduced 50 µM BAPTA-AM-evoked and 10 µM CPA-evoked single TRPC1 channel activities by >70% (Fig. 3*D* and Supplemental Fig. S3*A*). In contrast, bath application of 1 µM phorbol 12,13-dibutyrate, a direct PKC activator, to PLCβ1 knockdown VSMCs readily induced single TRPC1 channel activity, which indicates that PLCβ1 is involved in stimulation of TRPC1 SOCs upstream from PKC activity (Fig. 3*D*). These results provide clear evidence that PLCβ1 plays a major role in activation of TRPC1 SOCs in VSMCs.

### Store-depleted PLC activity is mediated by PLCβ1 isoform

Our results suggest that store depletion stimulates PLC activity mediated by PLCβ1. However, there is no previous evidence for store-operated PLC activity in VSMCs, and so we investigated this idea in more detail. Stimulation of PLC activity induces PIP_2_ hydrolysis at the plasma membrane to generate DAG and IP_3_, with the latter molecule diffusing into the cytosol. To monitor store-operated PLC activity in VSMCs, we transfected primary cultured VSMCs with GFP-PLCδ1-PH, a fluorescent biosensor with a high affinity for PIP_2_ and IP_3_ (30–33), and measured signal changes (in relative fluorescent units) at the plasma membrane [fluorescent intensity in membrane (Fm)] and within the cytosol [fluorescent intensity in cytosol (Fc)]. To provide a comprehensive analysis on whether store depletion induces PLC activity, we studied the effect of BAPTA-AM, CPA, and *N*,*N*,*N′*,*N′*-tetrakis(2-pyridylmethyl)ethane-1,2-diamineed (TPEN), a cell-permeable low-affinity Ca^2+^ chelator that selectively lowers Ca^2+^ levels within SR Ca^2+^ stores, on GFP-PLCδ1-PH signals.

**Figure 4.**
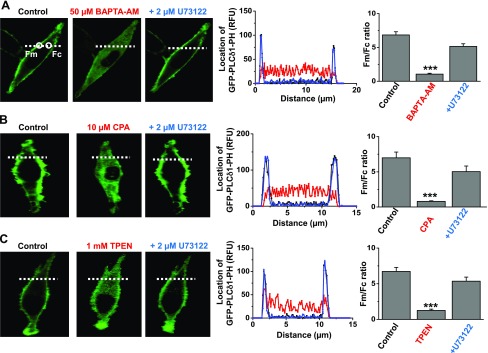
Store-depletion agents induce PLC activity. *A*) Representative image from a single cell shows that in control conditions, the location of GFP-PLCδ1-PH-mediated signals [measured in relative fluorescent units (RFU)] was predominantly expressed at the plasma membrane (black). In the same cell, pretreatment with BAPTA-AM induced translocation of signals to the cytosol (red), and coapplication of U73122 reversed these cytosolic signals back to the plasma membrane (blue). Graphs of relative fluorescence of line scans for the region denoted by white dotted lines show GFP-PLCδ1-PH signals across the cell width. Mean Fm:Fc ratios of GFP-PLCδ1-PH-mediated signals represent *n* = 20 cells from 3 different experiments. *B*, *C*) Data show that CPA (*B*) and TPEN (*C*) produced similar effects on GFP-PLCδ1-PH-mediated signals as BAPTA-AM (*n* = 20 cells from 3 experiments for each agent). ***P < 0.01.

In unstimulated cells, GFP-PLCδ1-PH signals were predominantly found located at the plasma membrane and had a mean Fm:Fc ratio of ∼7, which reflects the predominant cellular location of PIP_2_ and also suggests that there is limited cytosolic IP_3_ in these conditions (**Fig. 4**). Bath application of 50 µM BAPTA-AM, 10 µM CPA, or 1 mM TPEN for 10 min induced translocation of GFP-PLCδ1-PH signals from the plasma membrane to the cytosol, which relates to a reduction in mean Fm:Fc ratio of ∼80% (Fig. 4). These signal changes are likely to represent PLC-mediated PIP_2_ hydrolysis at the plasma membrane and subsequent generation of cytosolic IP_3_ (30–33). In support of these ideas, coapplication of 2 µM U73122 reversed BAPTA-AM-, CPA-, and TPEN-induced translocations of GFP-PLCδ1-PH signals (Fig. 4).

**Figure 5.**
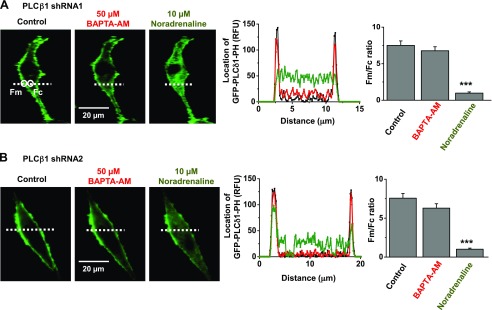
Store-operated PLC activity is mediated by PLCβ1. Representative images and mean data show that transduction of VSMCs with either PLCβ1 shRNA1 (*A*) or shRNA2 (*B*) sequences prevented BAPTA-AM (red) inducing translocation of GFP-PLCδ1-PH signals to the cytosol. In both these conditions, noradrenaline (green) was still able to induce translocation of GFP-PLCδ1-PH signals from the plasma membrane to the cytosol (*n* = 20 cells for each PLCβ1 shRNA sequence from 3 different primary cell culture preparations). ***P < 0.01.

**Figure 5** shows that PLCβ1 knockdown in VSMCs prevented translocation of GFP-PLCδ1-PH signals by 50 µM BAPTA-AM, whereas in the presence of scrambled shRNAs, BAPTA-AM induced similar effects on GFP-PLCδ1-PH signals as in Fig. 4 (data not shown). In comparison, stimulation of endogenously expressed α1 Gαq-coupled adrenoreceptors by bath application of 10 µM noradrenaline induced translocation of GFP-PLCδ1-PH signals from the plasma membrane to the cytosol in the presence of PLCβ1 shRNAs (Fig. 5). This indicates that other PLC isoforms, apart from PLCβ1, are likely to have a dominant role in mediating PLC activity induced by this concentration of noradrenaline. These results cannot exclude the possibility that noradrenaline-evoked PLCβ1 activity produces a small but irresolvable contribution to overall evoked PLC activity, which is involved in mediating stimulation of TRPC1 channels (Fig. 8*A*, *C*). These findings with noradrenaline also show that knockout of PLCβ1 does not have a general inhibitory effect on PLC activity, indicating that PLCβ1 shRNA is selective. Similar effects on GFP-PLCδ1-PH signals in the presence of PLCβ1 shRNAs were observed using 10 µM CPA and 1 mM TPEN (Supplemental Fig. S4).

We also investigated store-depletion–evoked PLC activity by measuring IP_3_ production using an ELISA. In primary cultured VSMCs, 10 µM noradrenaline induced an 8-fold increase in IP_3_ levels, which was prevented by pretreatment of 2 µM U73122 (Supplemental Fig. S3*B*). In comparison, 50 µM BAPTA-AM evoked over a 4-fold increase in IP_3_, which was also inhibited by 2 µM U73122 (Supplemental Fig. S3*B*).

Taken together, our findings provide strong evidence that store depletion induces PLCβ1 activity in VSMCs, which provides further support that Gαq-evoked PLC activity and PKC stimulation are important for activation of TRPC1 SOCs.

### Store depletion induces interactions between TRPC1, Gαq and PLCβ1

**Figure 6.**
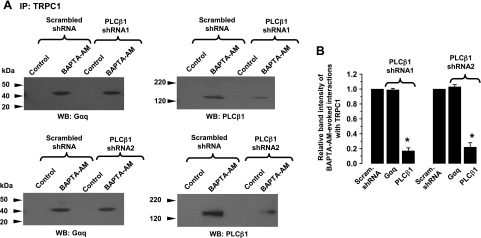
Store depletion evoked associations between TRPC1, Gαq and PLCβ1. *A*) Representative Western blots show that in unstimulated primary cultured rabbit portal vein VSMCs, TRPC1 did not associate with Gαq or PLCβ1. BAPTA-AM induced associations between TRPC1 and Gαq, and TRPC1 and PLCβ1, which were reduced by transduction of cells with either PLCβ1 shRNA1 or shRNA2 sequences. Primary cultured rabbit portal vein cell lysates initially immunoprecipitated (IP) with anti-TRPC1 antibodies were then Western blotted (WB) with anti-Gαq or anti-PLCβ antibodies. *B*) Mean data for relative band intensities of BAPTA-AM-evoked interactions between TRPC1 and Gαq or PLCβ1 (*n* = 3, different primary cell culture preparations). Scram., scrambled. *P < 0.05.

**Figure 7.**
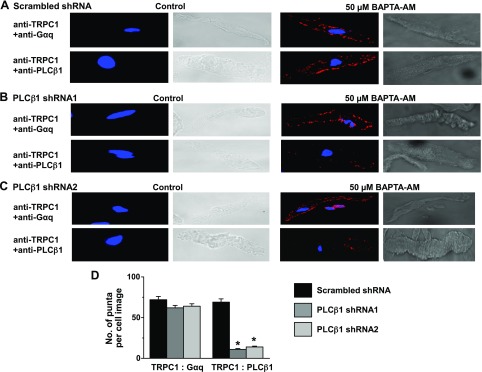
Store depletion induced colocalizations between TRPC1, Gαq and PLCβ1 at the plasma membrane. *A*) Representative PLA images from individual VSMCs show that BAPTA-AM induced fluorescent signals (red), which related to interactions between TRPC1 and Gαq, and TRPC1 and PLCβ1 in the presence of scrambled shRNA. *B*, *C*) Transduction of cells with PLCβ1 shRNA1 (*B*) and shRNA2 (*C*) sequences reduced BAPTA-AM-induced fluorescent signals between TRPC1 and PLCβ1 but did not alter those signals produced between TRPC1 and Gαq. *D*) Mean data show that both PLCβ1 shRNA1 and shRNA2 sequences prevented BAPTA-AM-induced punta formation between TRPC1 and PLCβ1 (*n* = 20, cells from 3 different experiments). *P < 0.05.

For store depletion to induce Gαq-evoked PLC activity and activate TRPC1 SOCs, it would seem appropriate that these molecules interact with one another, and therefore, we investigated these interactions using 2 techniques: coimmunoprecipitation, and proximity ligation assay. Immunoprecipitation with anti-TRPC1 antibodies followed by immunoblotting with either anti-Gαq or anti PLCβ1 antibodies failed to show any interactions between these molecules in unstimulated primary cultured cell lysates (**Fig. 6*A***). However, pretreatment of VSMCs with 50 µM BAPTA-AM for 10 min induced interactions between TRPC1 and Gαq, and between TRPC1 and PLCβ1 (Fig. 6*A*). Similar results were also obtained following pretreatment of freshly isolated vessel segments with 10 µM CPA (Supplemental Fig. S2*C*). As expected, transduction of VSMCs with PLCβ1 shRNAs significantly decreased BAPTA-AM-induced associations between TRPC1 and PLCβ1; however, PLCβ1 knockdown did not affect the interaction between TRPC1 and Gαq (Fig. 6). Proximity ligation assays showed no apparent signals between TRPC1 and Gαq, and TRPC1 and PLCβ1 in resting cells (**Fig. 7*A***), whereas pretreatment of cells with 50 µM BAPTA-AM for 10 min induced robust fluorescent signals (red) at the plasma membrane, which denoted interactions between TRPC1 and Gαq, and TRPC1 and PLCβ1 (Fig. 7*A*, *B*). These BAPTA-AM-evoked TRPC1-PLCβ1 signals were greatly reduced following transduction of VSMCs with PLCβ1 shRNAs, whereas BAPTA-AM-induced interactions between TRPC1 and Gαq remained unchanged (Fig. 7*B*, *C*, *D*). These findings clearly indicate that store depletion induces formation of TRPC1-Gαq-PLCβ1 complexes at the plasma membrane.

In control experiments, neither BAPTA-AM nor CPA altered expression levels of TRPC1, Gαq, PLCβ1, or PLCγ1, and neither one induced interactions between TRPC1 and PLCγ1 (Supplemental Fig. S2*C*). These negative results with PLCγ1 indicate that interactions between TRPC1 and PLCβ1 are selective.

### Noradrenaline-evoked TRPC1 activity requires PLCβ1

**Figure 8.**
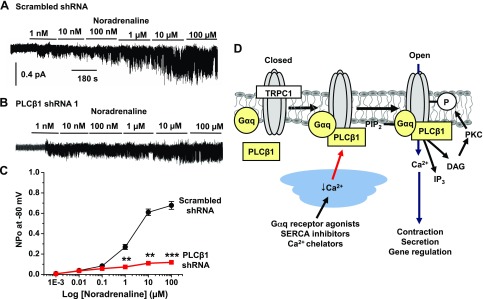
Proposed signal pathway coupling store depletion to activation of TRPC1 channels. *A*, *B*) Traces show that bath application of noradrenaline evoked TRPC1 channel activity in a concentration-dependent manner in cell-attached patches held at −80 mV, which were greatly reduced in VSMCs expressing PLCβ1 shRNA (*B*) compared to scrambled shRNA (*A*). *C*) Mean data show the inhibitory effect of PLCβ1 shRNA on noradrenaline-induced TRPC1 channel activity (*n* = 7). **P < 0.01; ***P < 0.001. *D*) Proposed activation model of TRPC1 channels in VSMCs. In the closed state, TRPC1 does not interact with Gαq and PLCβ1. Following Ca^2+^ store depletion, TRPC1 forms complexes with Gαq and PLCβ1 to cause PIP_2_ hydrolysis and formation of DAG, which stimulates PKC activity, phosphorylation of TRPC1 subunits, and channel opening.

Our above results clearly demonstrate that agents that deplete internal Ca^2+^ stores induce TRPC1 channel activity through a PLCβ1-mediated pathway. In our final experiments, we investigated whether a similar role for PLCβ1 is involved in mediating TRPC1 channel activity evoked by the physiologic agonist and vasoconstrictor noradrenaline. **Figure 8*A*, *C*** shows that bath application of noradrenaline (1 nM to 100 μM) activated 2 pS cation channel activity in a concentration-dependent manner in cell-attached patches held at −80 mV from VSMCs expressing scrambled shRNA. The properties of these channels are similar to TRPC1 channel activity previously recorded using store-depleting (see above) and vasoconstrictor agents (15, 21, 34, 35).

In the presence of PLCβ1 shRNA, noradrenaline-induced TRPC1 channel activity was greatly reduced (Fig. 8*B*, *C*). Interestingly, knockdown of PLCβ1 seemed to preferentially inhibit TRPC1 channel activity evoked by higher concentrations of noradrenaline (1–100 μM), whereas levels of channel activity evoked by lower concentrations were maintained (Fig. 8*B*, *C*).

These results strongly suggest that PLCβ1 has an important role in mediating TRPC1 channel activity induced by an endogenous agonist, which indicates the likely physiologic relevance of the proposed store-operated PLCβ1-mediated pathway in stimulating TRPC1 channels.

## DISCUSSION

The present work reveals for the first time that the classic phosphoinositol signaling pathway composed of Gαq-mediated PLCβ1 activity is stimulated by depletion of Ca^2+^ levels within SR Ca^2+^ stores in VSMCs. Moreover, store-operated Gαq-PLCβ1 activities coupled to PKC stimulation result in opening of TRPC1 SOCs. These results are likely to have widespread importance because phosphoinositol signaling and TRPC1 channels are ubiquitously expressed among cell types.

There is considerable evidence that SOCs are composed of a heteromeric TRPC1/C5 molecular template in contractile VSMCs; SOCs are absent in TRPC1^−/−^ VSMCs, reduced and increased by knockdown and overexpression of TRPC1 proteins, respectively, and inhibited by anti-TRPC1 and anti-TRPC5 antibodies (3, 14–22). In addition, TRPC1 and TRPC5 proteins colocalize with one another (21). These studies have provided considerable evidence that TRPC1 is the essential subunit that confers channel gating by store depletion, and therefore, these heteromeric TRPC1/C5 templates in VSMCs are often termed TRPC1 SOCs (22). The present work shows that well-established store-depletion agents with distinct mechanisms of action (*e.g.*, high intracellular BAPTA, BAPTA-AM, and CPA) activated whole-cell conductances with a relatively linear *I/V* relationship and an *E*_rev_ of ∼+20 mV, and also single-channel currents with a unitary conductance of ∼2 pS in freshly isolated and primary cultured VSMCs, which exhibit contractile phenotypes. Importantly, these studies did not observe SOCs in VSMCs, which had properties that resembled Orai1-mediated *I*_crac_ such as pronounced inward rectification and very positive *E*_rev_. Furthermore, our findings confirm that store-operated whole-cell and single-channel currents were inhibited by anti-TRPC1 antibodies. These results provide strong evidence that TRPC1 SOCs, and not Orai1-mediated *I*_crac_, are recorded in the present study.

Store-operated conductances with a linear *I/V* relationship and an *E*_rev_ of ∼0 mV were present in freshly isolated cerebral VSMCs from TRPC1^−/−^ mice (36), and inhibited by Orai1 small interfering RNA in contractile primary cultured mouse aorta VSMCs (37). There is currently no explanation why these 2 studies differ from the substantial number of studies, which indicate that SOCs in contractile VSMCs are mediated by TRPC1 channels. Recent studies suggesting that Orai1-mediated *I*_crac_ is expressed in long-term cultured VSMCs with synthetic or proliferative phenotypes may provide explanations (38, 39).

We have previously reported that PKC-dependent phosphorylation of TRPC1 proteins is obligatory for gating of TRPC1 SOCs in VSMCs (15, 17, 18, 22–25). In addition, PKCα-dependent phosphorylation of TRPC1 has also been reported to regulate store-operated Ca^2+^ entry in endothelial cells (40). However, it is not understood how store depletion is coupled to PKC stimulation, and therefore, this current study explored the possibility that store-operated Gαq-mediated PLC activity coupled to PKC is involved in opening of TRPC1 channels in VSMCs. Stimulation of store-operated whole-cell and single TRPC1 channel activities was prevented by G-protein, PLC, and PKC inhibitors. In addition, anti-Gαq/11 antibodies, but not by anti-Gαi1-3 antibodies, inhibited store-operated single TRPC1 channel activity, which implicates a role for Gαq/11 subunits in evoking TRPC1 SOCs. Moreover, PLC and PKC inhibitors reduced store-operated increases in phosphorylation of TRPC1 proteins. PLC and PKC inhibitors also reduced constitutive phosphorylation levels of TRPC1 proteins. Basal PKC phosphorylation may explain why TRPC1 SOCs are activated by agents such as PIP_2_, calmodulin, and MANS peptide in inside-out patches, which are unlikely to contain functional SR Ca^2+^ stores to drive Gαq-mediated PLC and PKC activities that are obligatory for channel gating (20, 25, 41). In future studies, it will be important to identify which PKC isoform is involved in gating TRPC1 channels and reveal which serine/threonine residues reported to be located at the putative pore region and N and C termini are involved (42).

To our knowledge, this is the first time that depletion of Ca^2+^ within SR Ca^2+^ stores has been proposed to induce Gαq-mediated PLC activity. Our data clearly show that the well-established store-depletion agents BAPTA-AM, CPA, and TPEN all induced translocation of GFP-PLCδ1-PH signals from the plasma membrane to the cytosol, which corresponds to stimulation of PLC activity, PIP_2_ hydrolysis, and production of IP_3_. In addition, store-operated changes in cellular distribution of GFP-PLCδ1-PH signals were reduced by a PLC inhibitor. GFP-PLCδ1-PH has previously been used to investigate changes in PIP_2_ and IP_3_ levels induced by stimulation of Gαq-coupled receptors and associated PLC-mediated signaling because it has a higher affinity for IP_3_ over PIP_2_ (30–33). In contrast, other agents that have much greater selectively for PIP_2_ over IP_3_ are useful for measuring changes in PIP_2_ levels regardless of PLC activity, such as Tubby (30–33). A rise in [Ca^2+^]_i_ may trigger PLC activity (33); however, this is unlikely to stimulate PLC activity in the present study because BAPTA-AM and TPEN, which reduce or have little effect on [Ca^2+^]_i_, respectively, induced translocation of GFP-PLCδ1-PH signals. Because BAPTA-AM, CPA, and TPEN deplete SR Ca^2+^ stores by such distinct actions, it is unlikely that the similar effects of these agents on GFP-PLCδ1-PH signals represent nonselective actions.

Transduction of primary cultured VSMCs with 2 distinct PLCβ1 shRNAs produced significant reductions in both store-operated whole-cell and single TRPC1 channel activities and also prevented store-operated translocation of GFP-PLCδ1-PH signals from the plasma membrane to the cytosol. These findings clearly show that the PLCβ1 isoform significantly contributes to store-operated PLC activity in VSMCs. PLCβ1 has also been linked to activation of TRPC channels in neurons (27–29). Previous studies have proposed that PLCγ1 has an important role in activation of TRPC1/C4-mediated SOCs in keratinocytes (43) and *I*_crac_-like currents in hepatocytes (44). It is thought that PLCγ1 enzymatic activity is not involved in activation of these channels; instead, PLCγ1 may act as a scaffold protein *via* its SH-2 domain (43, 44). These ideas are similar to those put forward for a role of PLCγ1 in agonist-induced Ca^2+^ entry (45). U73122 has also been shown to inhibit endogenous *I*_crac_-like currents and store-operated Ca^2+^ entry in RBL-2H3 cells (46). These studies further emphasize the novelty of the present work, that store-operated PLCβ1 enzymatic activity regulates TRPC1 SOCs.

Both coimmunoprecipitation studies and proximity ligation assays showed that store depletion induced interactions between TRPC1, Gαq and PLCβ1. Proximity ligation assays also identified that these interactions occurred at the plasma membrane and that they are likely to occur within 40 nm of each other (47).

PLCβ1 knockdown did not affect the associations between TRPC1 and Gαq, which suggests that these 2 interactions may occur as separate events during the formation of TRPC1-Gαq-PLCβ1 signaling complexes. Store depletion did not induce interactions between TRPC1 and PLCγ1, which suggests selective associations between TRPC1 and PLCβ1.

Our findings clearly show that TRPC1 channel activity induced by the endogenous Gαq-coupled receptor agonist and vasoconstrictor noradrenaline was prevented by knockdown of PLCβ1. This suggests that PLCβ1-mediated TRPC1 channel activation is likely to be physiologically important. Interestingly, reduction of noradrenaline-evoked TRPC1 channel activity by knockdown of PLCβ1 was most pronounced at concentrations of noradrenaline between 1 and 100 μM, which may suggest that these concentrations of noradrenaline are coupled to store depletion.

Taken together, our results indicate that in contractile VSMCs, depletion of Ca^2+^ within SR Ca^2+^ stores forms TRPC1-Gαq-PLCβ1 signaling complexes, which leads to increased PLCβ1 activity, production of DAG, and stimulation of PKC that induces TRPC1 channel gating (Fig. 8*D*). What is not yet understood is how store depletion induces formation of these complexes. A potential molecular candidate is STIM1, which is proposed to be involved in activation of overexpressed and endogenous TRPC1 channels through electrostatic and protein-protein interactions between STIM1 and TRPC1, including TRPC1 SOCs in VSMCs (8–13, 19, 38, 39, 48). In future experiments, it may be revealing to investigate if STIM1 and these STIM1 interaction sites mediate interactions with Gαq and PLCβ1, and also examine whether interactions between STIM1 and TRPC1 lead to dissociation of G proteins into Gαq and Gβγ subunits. It is increasingly apparent that STIM1 has diverse cellular partners, including ion channels such as Orai1 (4–7), TRPC channels (8, 12, 13) and voltage-gated Ca^2+^ channels (49, 50), SR and plasma membrane Ca^2+^-ATPases (51, 52), and adenylate cyclases (53). It will be intriguing to investigate if STIM1 coupled to Gαq-mediated PLC activity makes an important addition to this list, and also if Orai proteins have a role in these mechanisms.

We recently proposed an activation model of Gαq-coupled receptor-mediated TRPC1 channels in which interactions between TRPC1, MARCKS, PKC activity and PIP_2_ are obligatory partners in channel gating (25). Gαq receptor-mediated phosphorylation of TRPC1 by PKC induced dissociation of the PIP_2_-binding protein MARCKS from TRPC1 and also caused MARCKS to release PIP_2_, which then acted as a gating ligand (25). This finding suggested that MARCKS behaves as a reversible PIP_2_ buffer, providing a discrete pool of PIP_2_ for channel gating, which is protected from PLC-mediated PIP_2_ hydrolysis. It will be interesting to examine if store-operated TRPC1 channel activation by PKC involves similar roles for MARCKS and PIP_2_.

In conclusion, the present work proposes a novel gating pathway of TRPC1 channels; store depletion induces formation of TRPC1-Gαq-PLCβ1 complexes at the plasma membrane, which evoke Gαq-mediated PLC activity, PKC stimulation, and channel gating. Interestingly, this pathway will also generate IP_3_, which introduces the intriguing possibility that TRPC1-mediated Ca^2+^ entry, store refilling, and IP_3_-mediated store depletion produce discrete localized Ca^2+^ signals that selectively trigger cellular functions such as contraction, secretion, and gene regulation.

## Supplementary Material

Supplemental Data

## Abbreviations

BAPTA: 1,2-bis(2-aminophenoxy)ethane-*N,N,N′,N′*-tetraacetic acid
BAPTA-AM: 1,2-bis(2-aminophenoxy)ethane-*N,N,N′,N*′-tetraacetic acid acetoxymethyl ester
CPA: cyclopiazonic acid
DAG: diacylglyercol
Fc: fluorescent intensity in cytosol
Fm: fluorescent intensity in membrane
HEPES: 4-(2-hydroxyethyl)-1-piperazineethanesulfonic acid
IP_3_: inositol 1,4,5-trisphosphate
*I/V*: current-voltage
MARCKS: myristoylated alanine-rich C-kinase substrate
PIP_2_: phosphatidyinositol 4,5-bisphosphate
shRNA: small hairpin RNA
SOC: store-operated channel
SR: sarcoplasmic reticulum
TPEN: *N*,*N*,*N′*,*N′*-tetrakis(2-pyridylmethyl)ethane-1,2-diamineed
TRPC: canonical transient receptor potential
VSMC: vascular smooth muscle cell
