# Supplementary material for: Store depletion induces Gαq-mediated PLCβ1 activity to stimulate TRPC1 channels in vascular smooth muscle cells
Source: FASEB J. 2015 Oct 14;30(2):702–15. doi: 10.1096/fj.15-280271 (PMC4714548; doi:10.1096/fj.15-280271)
Supplement: Supplemental Data [file supp_30_2_702__index.html]

Store depletion induces Gαq-mediated PLCβ1 activity to stimulate TRPC1 channels in vascular smooth muscle cells — Store depletion induces Gαq-mediated PLCβ1 activity to stimulate TRPC1 channels in vascular smooth muscle cells — Store depletion induces Gαq-mediated PLCβ1 activity to stimulate TRPC1 channels in vascular smooth muscle cells — Supplemental Data 

# Store depletion induces Gαq-mediated PLCβ1 activity to stimulate TRPC1 channels in vascular smooth muscle cells

## Supplemental Data

- Supplemental Data
- Supplemental Data
- Supplemental Data
- Supplemental Data
