# Supplementary material for: Store depletion induces Gαq-mediated PLCβ1 activity to stimulate TRPC1 channels in vascular smooth muscle cells
Source: FASEB J. 2015 Oct 14;30(2):702–15. doi: 10.1096/fj.15-280271 (PMC4714548; doi:10.1096/fj.15-280271)
Supplement: Supplemental Data [file supp_fj.15-280271_Supplemental_Figures1.pdf]

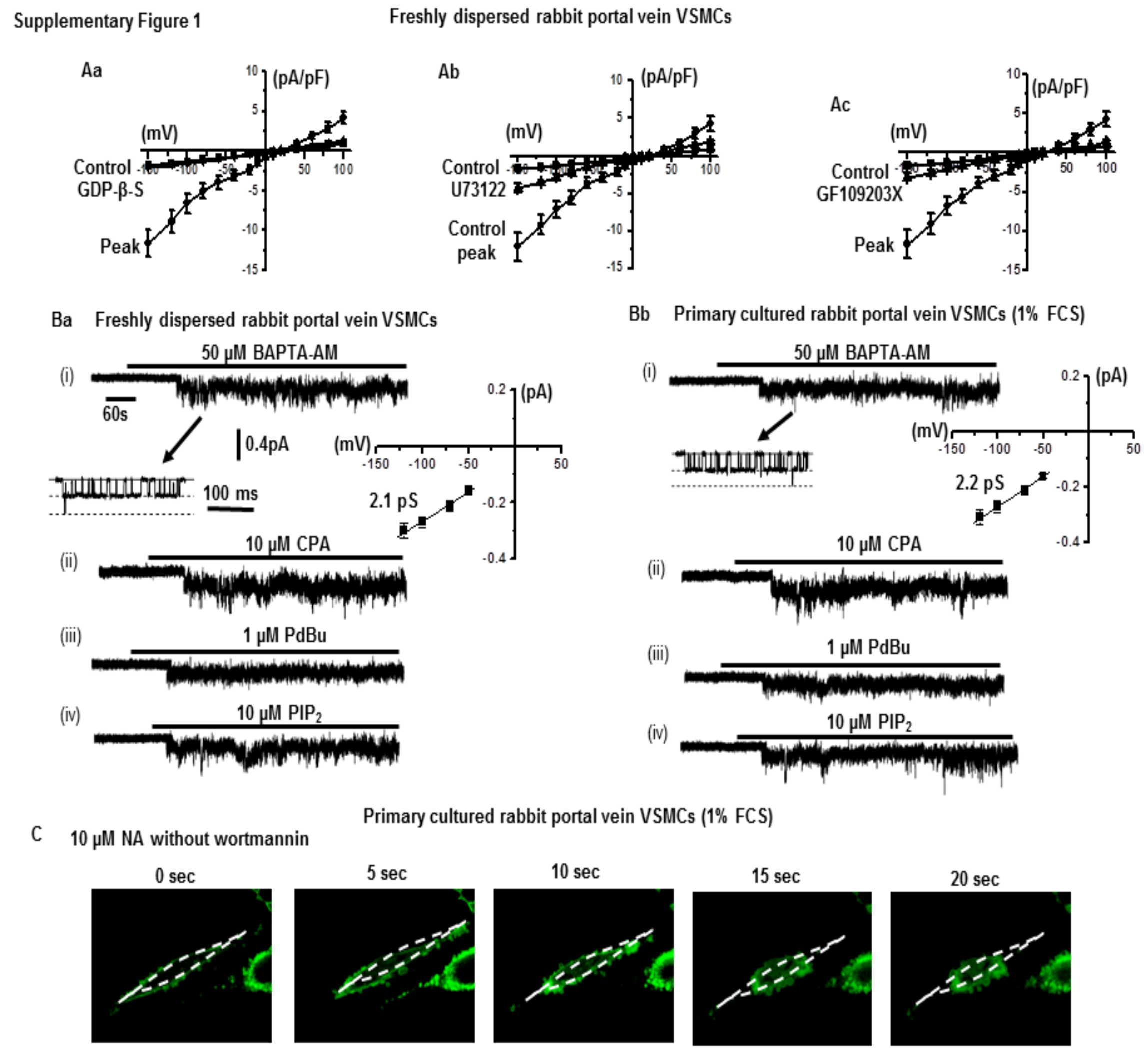

Figure S1. (**Aa**) Inclusion of 500 GDP-β-S in the patch pipette solution, or bath applications of (**Ab**) 2 μM U73122, and (**Ac**) 3 μM GF109203X inhibited store-operated whole-cell TRPC1 currents at all membrane tested (each point is from at least n=6). Note that peak whole-cell TRPC1 currents had relatively linear I/V relationships with E_{rev} of about +20 mV. (**Ba**) and (**Bb**) Bath applications of BAPTA-AM **(i)**, CPA **(ii)**, and PDBu **(iii)** activated single channel currents in cell attached patches held at -80 mV from freshly dispersed and primary cultured rabbit portal vein VSMCs. Insets of top traces show that BAPTA-AM-evoked single channel activities had unitary conductances of about 2 pS between -50 mV and -120 mV in both cell preparations. Bath application of the water-soluble PIP2 analogue, diC8-PIP2 **(iv)**, to the cytosolic surface of inside-out patches held at -80 mV also evoked similar single channel activities. (**C**) Time course images showing that application of noradrenaline (NA) in a wortmannin-free bathing solution induced contraction of single VSMCs expressing GFP-PLCδ-PH within 20 s. The white dotted line outlines the plasma membrane of the resting cell, which enables the noradrenaline-evoked contraction to be clearly visualised. In these cells, noradrenaline induced translocation of GFP-PLCδ-PH signals from the plasma membrane to the cytosol although accurate imaging of this translocation was precluded by the cell contracting.
