# Supplementary material for: Store depletion induces Gαq-mediated PLCβ1 activity to stimulate TRPC1 channels in vascular smooth muscle cells
Source: FASEB J. 2015 Oct 14;30(2):702–15. doi: 10.1096/fj.15-280271 (PMC4714548; doi:10.1096/fj.15-280271)
Supplement: Supplemental Data [file supp_fj.15-280271_Supplemental_Figures2.pdf]

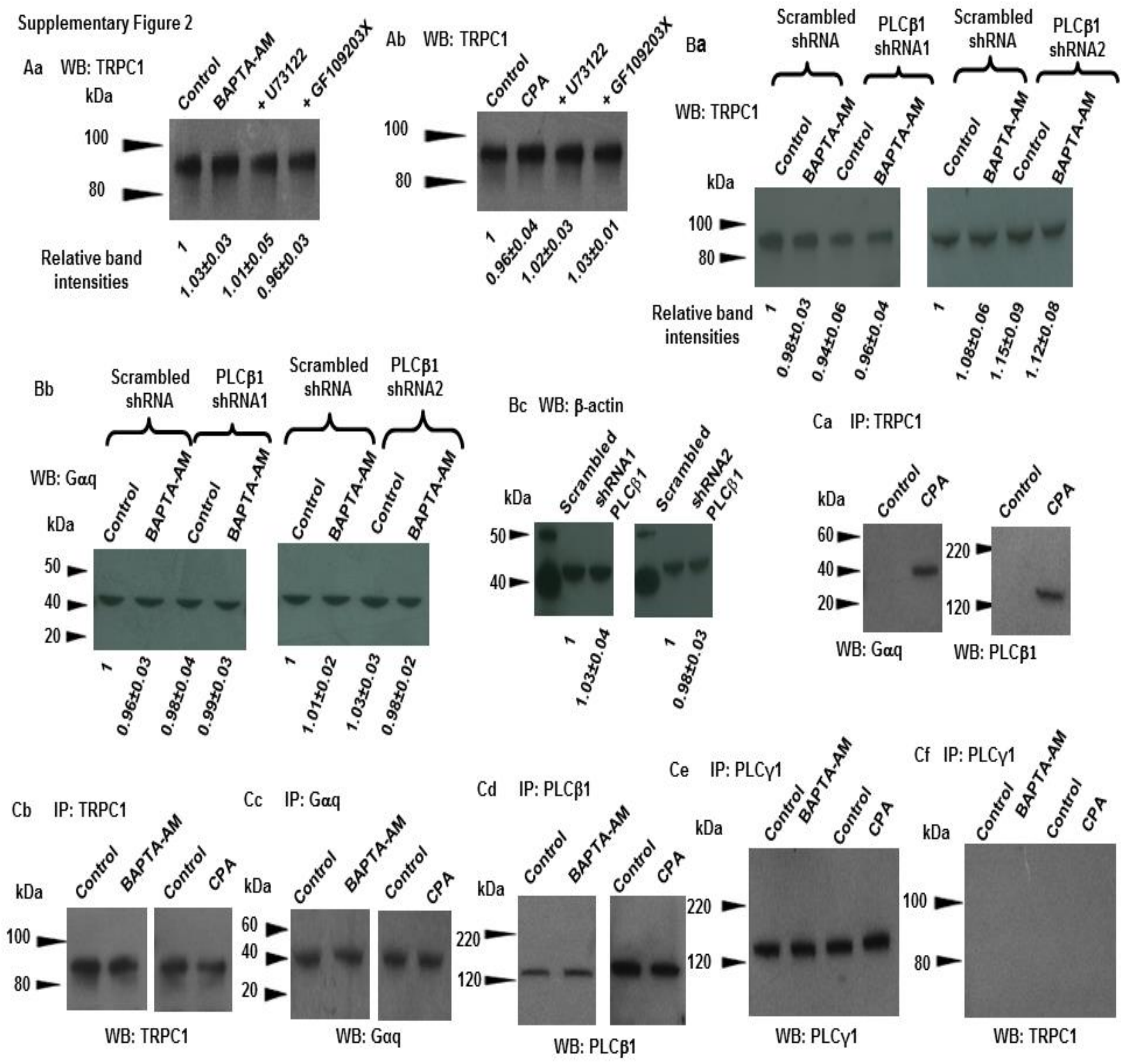

Figure S2. (**Aa**) and (**Ab**) Western blots showing that pre-treatment with BAPTA-AM or CPA alone, and co-application of U73122 or GF109203X did not change expression levels of TRPC1 proteins. Relative mean band intensities against control values are shown underneath blots (n=3 freshly isolated tissue lysates). (**Ba**) Western blots showing that scrambled shRNA, and PLCβ1 shRNA1 and shRNA2 sequences had no effect on expression of TRPC1 proteins (n=3 primary cultured cell lysates). (**Bb**) and (**Bc**) Western blots showing that PLCβ1 shRNA1 and shRNA2 sequences do not alter Gαq and β-actin expression levels (n=3 primary cultured cell preparations). (**Ca**) Co-immunoprecipitation with anti-TRPC1 antibodies followed by Western blotting with anti-Gαq or anti-PLCβ1 antibodies show that pre-treatment of vessel segments with CPA induced interactions between TRPC1 and these molecules. (**Cb**) to (**Cd**) Pre-treatment of vessels with BAPTA-AM or CPA did not alter expression levels of TRPC1, Gαq, and PLCβ1. (**Ce**) and (**Cf**) Pre-treatment with BAPTA-AM or CPA also had no effect on expression levels of PLCγ1 and co-immunoprecipitation with anti-PLCγ1 antibodies followed by Western blotting with anti-TRPC1 antibodies showed no interactions between these two molecules at rest or following pre-treatment with BAPTA-AM or CPA.
