# Supplementary material for: Store depletion induces Gαq-mediated PLCβ1 activity to stimulate TRPC1 channels in vascular smooth muscle cells
Source: FASEB J. 2015 Oct 14;30(2):702–15. doi: 10.1096/fj.15-280271 (PMC4714548; doi:10.1096/fj.15-280271)
Supplement: Supplemental Data [file supp_fj.15-280271_Supplemental_Figures3.pdf]

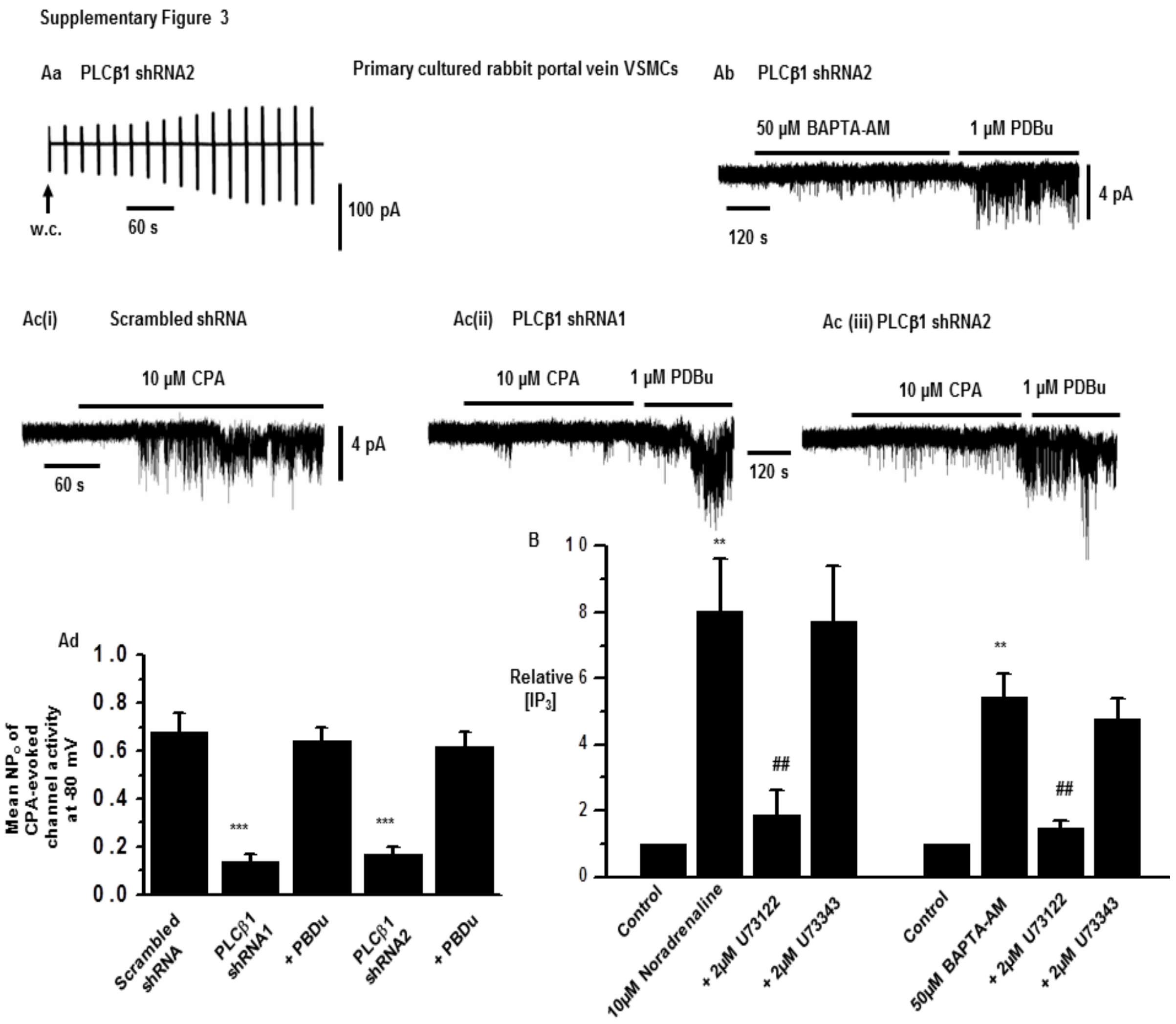

Figure S3. (**Aa**) and (**Ab**) Representative traces showing that transduction of VSMCs with PLCβ1 shRNA2 produced greatly reduced store-operated whole-cell currents and TRPC1 channel activity (see Fig. 3 for mean data). (**Ac**) and (**Ad**) Representative recordings and mean data showing that CPA-evoked TRPC1 SOC activities were reduced by PLCβ1 shRNA1 and shRNA2 compared to scrambled shRNA but this did not affect channel activation by PDBu (n=7, ****P*<0.001). (**B**) Pre-treatment with noradrenaline induced an approximate 8-fold increase in relative IP_3 level measured using an ELISA kit, which was prevented by co-application of U73122 but not by U73343. BAPTA-AM induced over a 4-fold increase in relative IP_3 level which was also prevented by U73122 but not by U73343. (n=3 cultured cell preparations, **P<0.01 (vs. control), $^{\#\#}$P<0.01 (NA- or BAPTA-AM-evoked increase in IP_3 levels vs. U73122).
