# Supplementary material for: Store depletion induces Gαq-mediated PLCβ1 activity to stimulate TRPC1 channels in vascular smooth muscle cells
Source: FASEB J. 2015 Oct 14;30(2):702–15. doi: 10.1096/fj.15-280271 (PMC4714548; doi:10.1096/fj.15-280271)
Supplement: Supplemental Data [file supp_fj.15-280271_Supplemental_Figures4.pdf]

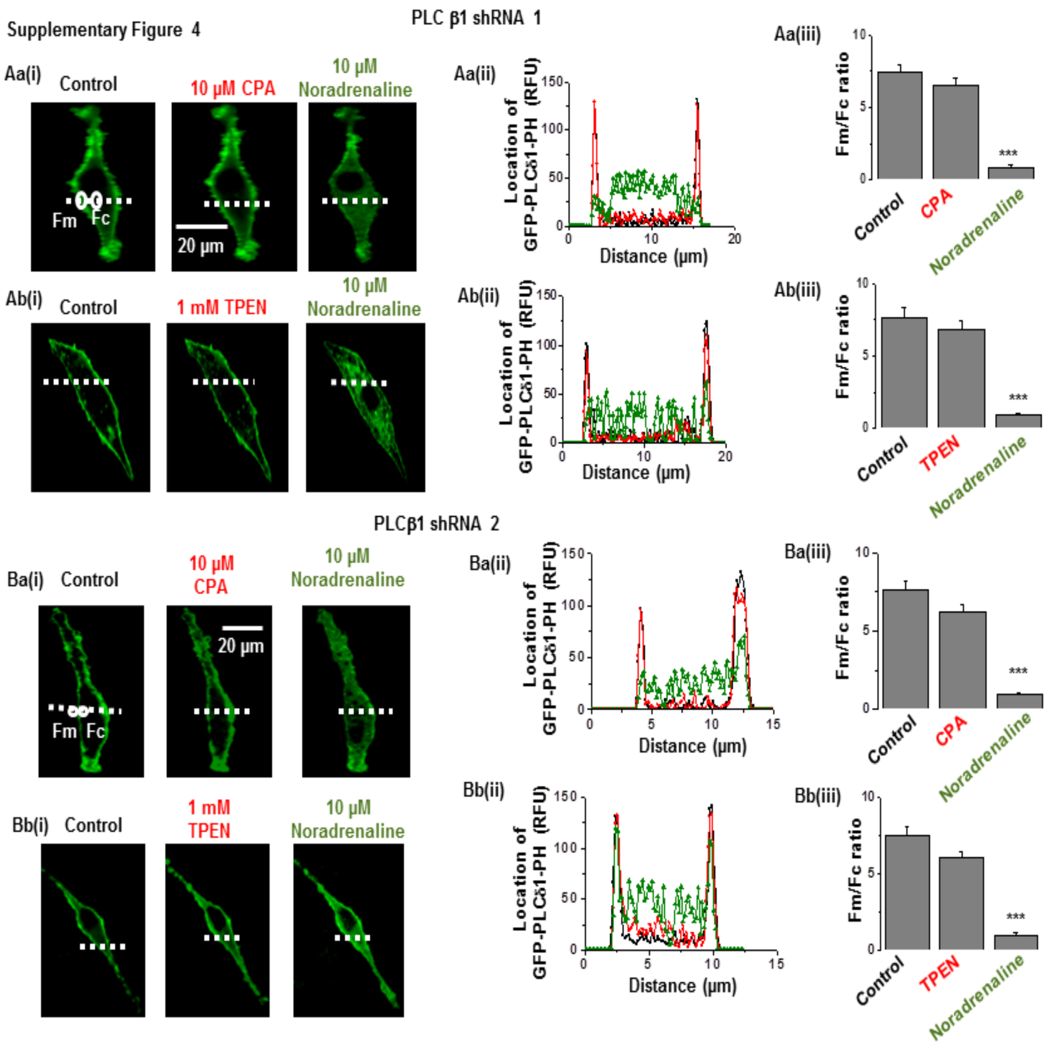

Figure S4. (**Aa**) and (**Ab**) Representative images showing that BAPTA-AM and TPEN failed to induce translocation of GFP-PLCδ1-PH signals to the cytosol (red) following transduction of VSMCs with PLCβ1 shRNA1. Noradrenaline (green) was still able to induce translocation of GFP-PLCδ1-PH signals from the plasma membrane to the cytosol in the presence of PLCβ1 shRNA1. Mean data from n=20 cells from three primary cell culture preparations for CPA and TPEN, **P<0.01. (**Ba**) and (**Bb**) Representative images showing that BAPTA-AM and TPEN failed to induced translocation of GFP-PLCδ1-PH signals to the cytosol (red) following transduction of VSMCs with PLCβ1 shRNA2. Noradrenaline (green) was still able to induce translocation of GFP-PLCδ1-PH signals from the plasma membrane to the cytosol in the presence of PLCβ1 shRNA2. Mean data from n=20 cells from three primary cell culture preparations for CPA and TPEN, ***P<0.01.
